# Exploring potential molecular resistance and clonal evolution in advanced HER2-positive gastric cancer under trastuzumab therapy

**DOI:** 10.1038/s41389-023-00466-2

**Published:** 2023-04-18

**Authors:** Qi Xu, Xiaoqing Xu, Haimeng Tang, Junrong Yan, Jingjing Li, Hua Bao, Xue Wu, Yang Shao, Cong Luo, Haimin Wen, Jianying Jin, Jieer Ying

**Affiliations:** 1 Department of Hepato-Pancreato-Biliary & Gastric Medical Oncology, Zhejiang Cancer Hospital, 310022 Hangzhou, China; 2 Institute of Basic Medicine and Cancer (IBMC), Chinese Academy of Sciences, 310022 Hangzhou, China; 3 Department of Medical Oncology, The Second Clinical Medical College of Zhejiang Chinese Medical University, 310053 Hangzhou, China; 4 Geneseeq Research Institute, Nanjing Geneseeq Technology Inc., 210031 Nanjing, China; 5 School of Public Health, Nanjing Medical University, 211166 Nanjing, Jiangsu China; 6 Department of Medical Oncology, Taizhou Hospital of Zhejiang Province, 317000 Taizhou, China; 7 Key Laboratory of Prevention, Diagnosis and Therapy of Upper Gastrointestinal Cancer of Zhejiang Province, 310022 Hangzhou, China

**Keywords:** Gastric cancer, Targeted therapies

## Abstract

HER2-positive gastric cancer (GC) makes up 15–20% of all GC incidences, and targeted therapy with trastuzumab is the standard of treatment. However, the mechanisms of resistance to trastuzumab are still not fully understood and presents a significant challenge in clinical practice. In this study, whole exome sequencing (WES) was performed on paired tumor tissues before trastuzumab treatment (at baseline) and at progressive disease (PD) in 23 GC patients. Clinicopathological and molecular features that may be associated with primary and/or acquired resistance to trastuzumab were identified. Lauren classification of intestinal type was associated with a more prolonged progression-free survival (PFS) than diffuse type (HR = 0.29, *P* = 0.019). Patients with low tumor mutation burden (TMB) showed significantly worse PFS, while high chromosome instability (CIN) was correlated with prolonged OS (HR = 0.27; *P* = 0.044). Patients who responded to treatment had a higher CIN than nonresponders, and a positive trend towards increasing CIN was observed as response improved (*P* = 0.019). In our cohort, the most common genes to acquire mutations are *AURKA, MYC, STK11*, and *LRP6* with four patients each. We also discovered an association between clonal branching pattern and survival, with an extensive clonal branching pattern being more closely related to a shorter PFS than other branching patterns (HR = 4.71; *P* = 0.008). We identified potential molecular and clinical factors that provide insight regarding potential association to trastuzumab resistance in advanced HER2-positive GC patients.

## Introduction

Gastric cancer (GC) is the fifth most common cancer and the fourth leading cause of cancer-related deaths worldwide [1, 2]. In China, most patients are diagnosed at advanced disease and therefore will not undergo surgery as part of the routine treatment. Currently, the mainstream treatment for unresectable or metastatic GC involves the use of chemotherapy, but the prognosis remains poor.

Human epidermal growth factor receptor 2 (HER2) [3, 4] is a transmembrane protein coded by the *ERBB2* gene. HER2-positive GC is when overexpression of HER2 proteins is observed on the surface of the tumor [3]. HER2-positive gastric cancers account for approximately one-quarter of all newly diagnosed GC. Trastuzumab, a monoclonal antibody targeting HER2, has been shown to increase survival benefit in combination with chemotherapy [5, 6]. In the highly influential ToGA study [7], it was shown that compared with chemotherapy alone, the application of targeted therapy with trastuzumab in combination with chemotherapy could prolong the progression-free survival (PFS) of patients with HER2-positive GC from 5.5 to 6.7 months, while simultaneously improving the objective response rate (ORR) from 35 to 47%. However, only about half of HER2-positive GC patients were responsive to trastuzumab, and primary resistance was observed in a subset of patients [7].

Primary or acquired resistance to trastuzumab is the cause of most treatment failures. Several potential mechanisms of drug resistance have been previously reported [8–12]: (1) HER2 heterogeneity; (2) loss of HER2 positivity/acquired HER2 mutations; (3) HER2 heterodimers; (4) altered intracellular signaling. Currently, resistance to trastuzumab remains a major obstacle that limits clinical benefit. Therefore, identifying the potential key genes that invoke resistance to trastuzumab can differentiate between sensitivity subgroups to improve the overall treatment of GC.

The design of this study was influenced by a paper published in Gut in 2018 by Wang et al. [13]. In their study, plasma samples of HER2-positive GC patients were tracked longitudinally to determine trastuzumab resistance mechanisms. Currently, liquid biopsy is the mainstream approach in clinical practice for dynamically tracking HER2 resistance [13–15], while the more invasive tissue biopsy is seldom used. However, compared to tissue biopsy, circulating tumor DNA is less sensitive to mutational changes and are more prone to both false positive and false negative results. Therefore, tissue biopsy is considered the more reliable way to investigate tumor genotypes. Unfortunately, most patients do not receive surgery as a standard-of-care procedure for metastatic GC, making it difficult to obtain sufficient samples within a reasonable time frame.

In this study, we compared the genomic landscape of stage IV GC patients with paired tumor tissue samples before trastuzumab treatment (at baseline) and at progressive disease (PD) to identify possible primary and/or acquired mechanisms of trastuzumab resistance. We were able to extract samples from the same tumor site for each patient at two timepoints, one at baseline and one at progressive disease, allowing us to make a more reliable comparison by minimizing the heterogeneous effect of genetic variation between samples. By using tumor tissue samples, we can ensure that most, if not all, captured DNA originated from the tumor site, leading to a more accurate detection of relevant somatic mutations. Through this investigation, we sought to identify potential biomarkers related to primary resistance and understand what molecular alterations may be associated with the acquired resistance of trastuzumab. The results of this study would provide insight on the likely mechanisms of action for either of these resistances in patients.

## Results

### Patient characteristics

**Fig. 1:**
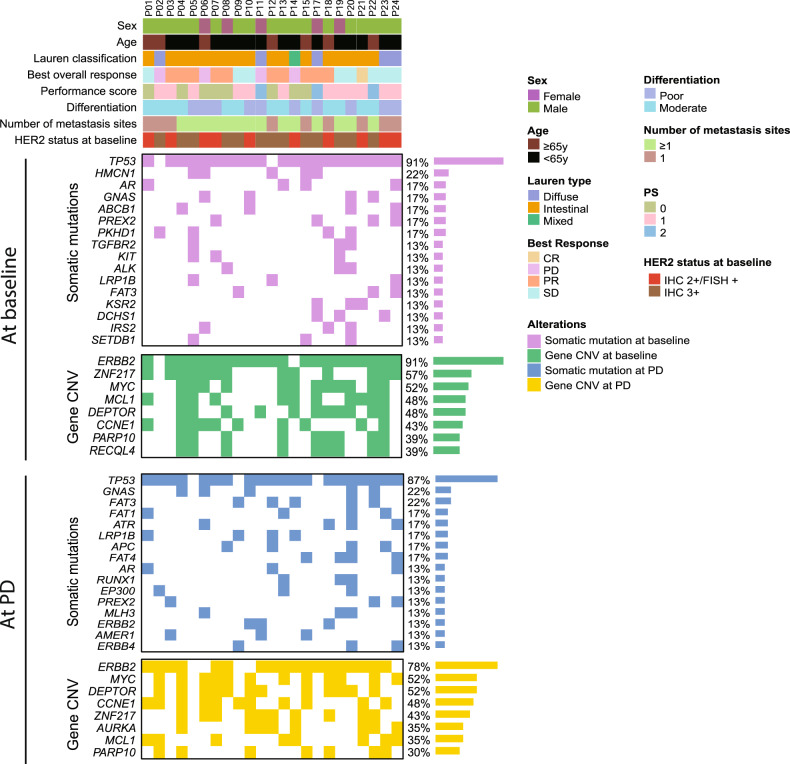
Mutational landscape for 23 GC patients at baseline and at PD. Only top sixteen gene with somatic mutations and top eight genes with CNVs were shown. Genes were ordered according to the frequency of appearance. CR complete response, PR partial response, SD stable disease, PD progressive disease, CNV copy number variation, IHC immunohistochemistry, FISH fluorescence in situ hybridization.

A total of 24 GC patients with HER2-positive receiving trastuzumab combination therapy were included in the study cohort, and their baseline clinicopathological characteristics were summarized and presented in Table 1 and Fig. 1 (median age 63 years, range 21–73; 79.2% of patients were male). According to the Lauren classification, 18 (75%) patients were intestinal type, five patients (20.8%) were diffuse type, and one patient (4.2%) was the mixed type. Of all the patients, 14 (58.3%) had moderately differentiated tumors, and 10 (41.7%) had poorly differentiated tumors. Common metastatic organs were liver and lymph node, which were found in 15 (62.5%) patients and 16 (66.7%) patients, respectively. 21 patients (87.5%) had a performance status of one or lower, and three patients (12.5%) had a performance status of two or more. Using IHC, we differentiated between the patients based on their HER2 status at baseline. 14 (58.3%) showed IHC3+ and 10 (41.7%) showed IHC2+ and FISH + . All patients received trastuzumab in combination with chemotherapy as a first-line treatment strategy.

**Table 1:** Clinicopathologic characteristics of the 24 GC patients enrolled in this study.

Characteristic	Total (*n* = 24)	Primary resistance (*n* = 4)	Acquired resistance (*n* = 20)
Age, median (range)	63 (21–73)	60.5 (47–71)	63 (21–73)
Age, no. (%)			
≥65	8 (33.3)	2 (50)	6 (30)
<65	16 (66.7)	2 (50)	14 (70)
Sex, no. (%)			
Male	19 (79.2)	2 (50)	17 (85)
Female	5 (20.8)	2 (50)	3 (15)
ECOG PS			
0–1	21 (87.5)	3 (75)	18 (90)
2	3 (12.5)	1 (25)	2 (10)
Lauren type, no. (%)			
Diffuse	5 (20.8)	2 (50)	3 (15)
Intestinal	18 (75)	1 (25)	17 (85)
Mixed	1 (4.2)	1 (25)	0 (0)
Treatment line, no. (%)			
First line	24 (100)	4 (100)	20 (100)
Differentiation, no. (%)			
Moderate	14 (58.3)	1 (25)	13 (65)
Poor	10 (41.7)	3 (75)	7 (35)
Metastasis location, no. (%)			
=1	9 (37.5)	1 (25)	8 (40)
≥2	15 (62.5)	3 (75)	12 (60)
Organ of metastasis, no. (%)			
Lymph node	16 (66.7)	3 (75)	13 (65)
Peritoneal	5 (21.7)	1 (25)	4 (20)
Liver	15 (62.5)	4 (100)	11 (55)
Initial HER2 status verification, no. (%)			
IHC3 +	14 (58.3)	2 (50)	12 (60)
IHC2 + /FISH positive	10 (41.7)	2 (25)	8 (40)
Best response, no. (%)			
PD	4 (16.6)	4 (100)	0 (0)
SD	9 (37.5)	0 (0)	9 (45)
PR	10 (41.7)	0 (0)	10 (50)
CR	1 (4.2)	0 (0)	1 (5)

### Efficacy of combination therapy

As of July 6, 2021, 24 patients (100%) had experienced progression, and 11 (45.8%) had died. The median PFS (mPFS) and median overall survival (mOS) were, respectively, 7.2 months and 12.6 months (Supplemental Fig. S2A, B). Overall, the ORR was 45.8% and the disease control rate (DCR) was 83.3%. Among the 23 patients with whole exome sequencing (WES) data, four were identified as having primary resistance (P02, P06, P11, P14). One patient without matching WES data was removed from genetic analyses. Of the acquired resistance patients, there was only one (5.3%) patient with complete response (CR), 10 (52.6%) with partial response (PR), and eight (42.1%) with stable disease (SD) (Supplemental Fig. S1).

### Concordance of next-generation sequencing methods and clinical validation of HER2 status

HER2 status of all patients was observed using IHC and validated by FISH (Supplemental Fig. S3 and Supplemental Table S1). Through clinical validations, we did not find any significant difference between the PFS or OS of patients with IHC2 + /FISH + and IHC3 + (Supplemental Fig. S3A–C). Next, we used FACETS to call copy number variations (CNV) of the ERBB2 gene. Next-generation sequencing (NGS) approaches show that ERBB2 amplification was absent in two patients (P02 and P14). Both patients were classified as having primary resistance to trastuzumab, and the difference between groups was determined to be significant (Fisher’s exact test *P* = 0.022, Supplemental Fig. S3D). Supplemental survival analysis of the NGS-differentiated groups showed significant differences in both PFS and OS. We calculated a mPFS of 1.43 months for the two samples without NGS-detected ERBB2 amplifications and 7.83 months for the samples with NGS-detected ERBB2 amplifications (Supplemental Fig. S3E). For overall survival, the median OS for the group with NGS-detected ERBB2 amplification was more than eight times higher than that of the others (mOS_amplification_ vs. mOS_WT_: 38.30 months vs. 4.45 months, *P* < 0.0001, Supplemental Fig. S3F).

With results from NGS methods and IHC/FISH, we could then assess the concordance between the two methods. Two patients who were deemed to be HER2-positive through IHC/FISH were found not to have ERBB2 amplification through sequencing. This discrepancy has been reported previously in a paper by Niu et al. in 2020, in which they made a claim that, for GC patients, NGS methods of determining HER2 status have a lower sensitivity when compared to IHC/FISH [16].

### Baseline molecular analysis and clinical factors reveal potential indicators of primary resistance to trastuzumab treatment

**Fig. 2:**
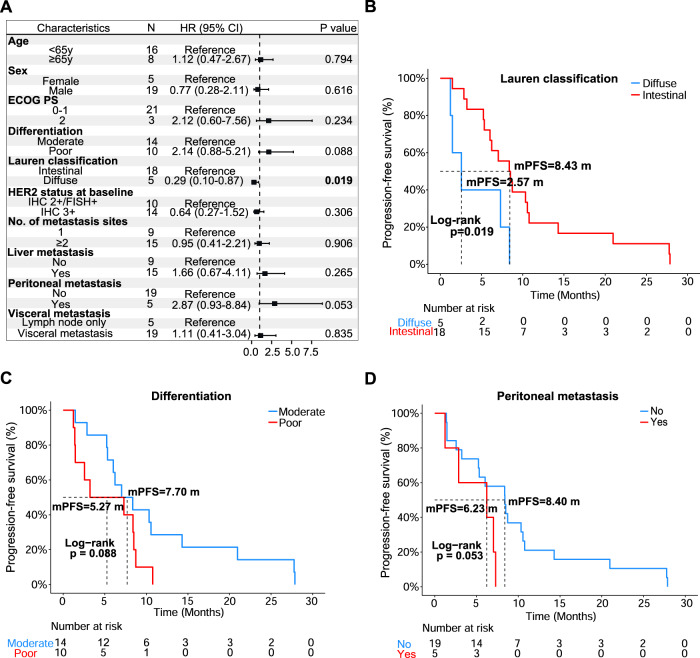
Cox proportional hazard model for clinical factors. **A** Forest plot for clinical factors. The characteristic used for comparison was labeled “Reference”. A black box denotes the mean hazard ratio for each comparison to the reference. Error bars on forest plots represent the 95% confidence interval for each hazard ratio. Significant *P* values were shown in bold. Kaplan–Meier curves were shown for **B** Lauren classification, **C** differentiation, and **D** peritoneal metastasis. Log-rank test was used to determine significance. ECOG PS Eastern Cooperative Oncology Group performance status.

The landscape of molecular alterations for our cohort is shown in Fig. 1. Somatic mutations in *TP53* were the most common, being altered in 21 (91%) patients. For CNVs, *ERBB2* amplifications were the most common (91%, 21/23). We then analyzed the correlation between clinicopathological characteristics and treatment outcomes. Features including age, sex, performance status, number of metastasis organs, liver metastasis events, lymph node metastasis events, and initial HER2 status did not significantly influence survival (Fig. 2A).

On the other hand, Lauren classification was found to be significantly correlated with a worse PFS. According to the Lauren classification, patients with intestinal phenotypes were less susceptible to disease progress (mPFS: 8.43 vs. 2.57 months; HR = 0.29, 95% CI = 0.10–0.87; *P* = 0.019; Fig. 2B) than those with diffuse phenotypes. No significant difference in OS was seen in these patients (Data not shown). In addition, we noted that patients with poorly differentiated tumors tended to have worse PFS (mPFS: 7.70 vs. 5.27 months; HR = 2.14, 95% CI: 0.88–5.21; *P* = 0.088; Fig. 2C) than those with moderately differentiated tumors. In the event of organ metastases, we found that patients with peritoneal metastases showed a worse trend for PFS than those who had no peritoneal metastasis (mPFS: 8.40 vs. 6.23 months; HR = 2.87, 95% CI: 0.93–8.84; *P* = 0.053; Fig. 2D).

**Fig. 3:**
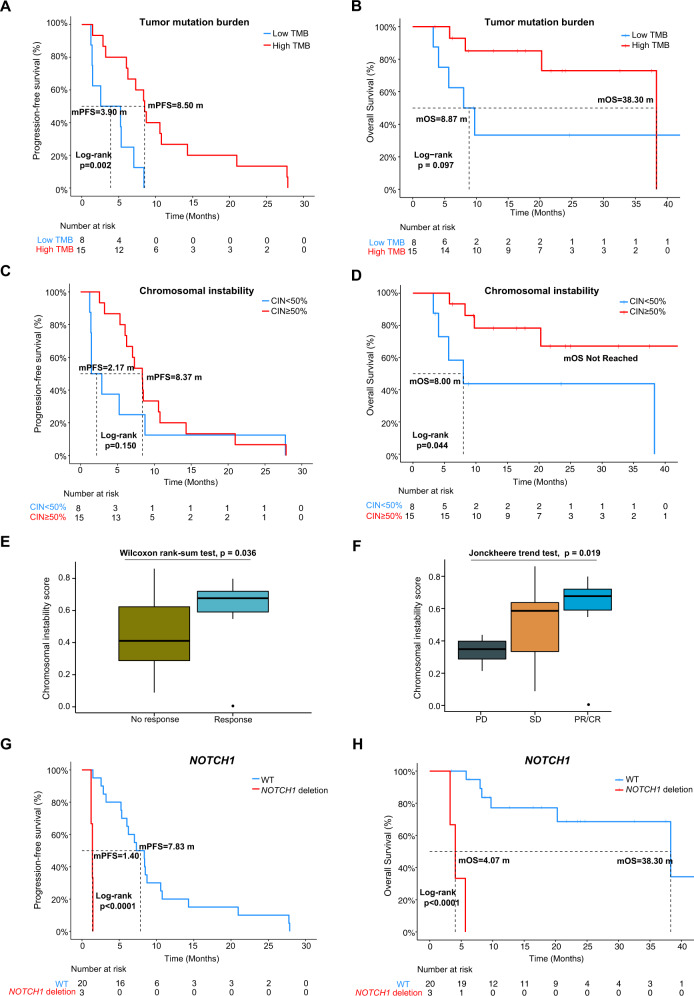
Characteristic and significant alterations for 23 patients at baseline. Survival analysis of TMB for patients with **A** PFS or **B** OS as endpoint. **C** Survival analysis for patients with high and low CIN with PFS or **D** OS as endpoint. **E** Boxplot of CIN distribution for response and no response patients. Wilcoxon ranked sum test was used to determine significance. **F** Trend test for significance of CIN across different response groups. Two-tailed Jonckheere’s trend test was used to determine the significance. **G** Kaplan–Meier curve of a patient with *NOTCH1* deletion using PFS and **H** OS as endpoint. CR complete response, PR partial response, SD stable disease, PD progressive disease, WT wild type, TMB tumor mutation burden, CIN chromosomal instability.

To investigate sample-level characteristics and how they correlate to treatment response, we first focused on TMB. We divided the patients into low TMB (*N* = 8) and high TMB (*N* = 15) of groups based on a cutoff at one-third of the total samples. Interestingly, we observed that patients with low TMB were more likely to experience PD (mPFS: 3.90 vs. 8.50 months; HR = 4.63, 95% CI: 1.62–13.20; *P* = 0.002, Fig. 3A, Supplemental Table S1) and OS was visibly shorter but not statistically different to high TMB patients (mOS: 8.87 vs. 38.30 months; HR = 2.95, 95% CI: 0.78–11.20; *P* = 0.097, Fig. 3B, Supplemental Table S1). Further comparisons of sample distribution showed that there was no significant difference between low and high TMB groups regardless of which categories were chosen (Fisher’s exact test, *P* = 0.103 and *P* = 0.193, Supplemental Fig. S4A, B, Supplemental Table S1).

Another sample-level metric that was analyzed is chromosomal instability (CIN). Using 50% CIN as a cutoff, we split the 23 patients into a “high instability” group (*N* = 15) and a “low instability” group (*N* = 8). We discovered that, while PFS did not significantly differ between the groups (mPFS: 8.37 vs. 2.17 months; HR = 0.53, 95% CI: 0.22–1.28; *P* = 0.150), the group with lower CIN had a worse OS than the high CIN group (mOS: Not reached vs. 8.00 months; HR = 0.27, 95% CI: 0.07–1.04; *P* = 0.044) (Fig. 3C, D and Supplemental Table S1). This result was corroborated when the clinical response was considered, and we found that, surprisingly, the patients who responded well to treatment had a higher CIN than those who did not respond well (# of CR + PR vs # of SD + PD; Wilcoxon rank-sum test, *P* = 0.036, Fig. 3E). When the groups were further split, a distinct trend towards higher CIN was observed as response improved (Jonckheere’s two-tailed trend test, *P* = 0.019, Fig. 3F). All four primary resistance patients had chromosomal instability scores lower than 50% (Fisher’s exact test, *P* = 0.008, Supplemental Fig. S4C, Supplemental Table S1). The distribution of response and no response patients in high CIN and low CIN was also found to be significantly different (Fisher’s exact test, *P* = 0.027, Supplemental Fig. S4D, Supplemental Table S1).

Finally, determining the distribution of diffuse and intestinal Lauren classification of GC patients showed no significant differences between grouping categories (Fisher’s exact test, *P* = 0.107 and *P* = 0.317, Supplemental Fig. S4E, F).

Besides the sample-level markers listed above, we also investigated gene-level molecular alterations that were present in samples at baseline. While no somatic mutations were significantly correlated with PFS or OS, some gene CNVs were found to be significantly associated with these endpoints (Supplemental Tables S2 and S3). *NOTCH1* deletion was particularly noteworthy, as it was only detected in patients with primary resistance at baseline. Survival analysis for this alteration showed that the difference between wild-type (WT) and *NOTCH1* deletion patients was highly significant for both PFS (mPFS: 1.40 vs. 7.83 months; HR = 51.00, 95% CI: 4.80–540.00; *P* < 0.0001) and OS (mOS: 4.07 vs. 38.30 months; HR = 1 × 10^10^, 95% CI: 0-infinity; *P* < 0.0001) (Fig. 3G, H). Both Fisher’s test results showed that there was significant difference between the distribution of patients in each category (Supplemental Fig. S4G, H).

To summarize the findings at baseline analysis, patients with poorer response to trastuzumab treatment were found to share common features such as having a diffuse Lauren classification, low CIN, and baseline *NOTCH1* deletion, which suggests that these factors may be indicative of primary trastuzumab resistance in patients.

### Patients with acquired resistance to trastuzumab treatment tend to acquire *ERBB4* and *FAT4* mutations

**Fig. 4:**
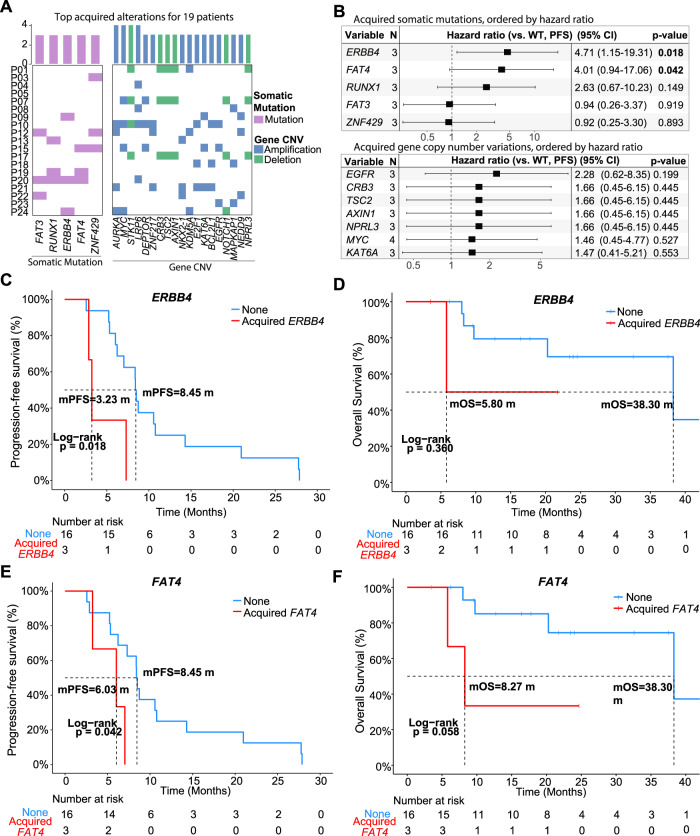
Acquired mutations and CNVs for 19 patients with acquired resistance to trastuzumab. **A** Oncoprint for acquired mutations and CNVs across 19 patients with acquired resistance. Only alterations with ≥3 supporting samples were selected to be shown. **B** Forest plot for the hazard ratio of patients who acquired somatic mutations (Top) or CNVs (Bottom) vs. WT based on their PFS. A black box denotes the mean hazard ratio for each comparison to the reference. Error bars on forest plots represent the 95% confidence interval for each hazard ratio. Significant *P* values were shown in bold. Log-rank test was used to test for significance. **C** Survival curve of *ERBB4* mutations based on PFS or **D** OS of patients. Kaplan–Meier curves for acquired *FAT4* mutations were shown according to **E** PFS or **F** OS. WT wild type, CNV copy number variation.

To examine the acquisition of alterations by patients who developed resistance to trastuzumab, we compared the genomic landscape of samples from before treatment and after disease progression. Four patients with primary resistance were removed from this analysis. Acquired alterations for 19 samples show that the most common acquired alterations (i.e., not present in matching baseline sample) were *AURKA* amplification (4/19), *MYC* amplification (4/19), *STK11* deletion (4/19), *LRP6* amplification (4/19) (Fig. 4A). No CNVs were deemed to be significantly correlated with PFS or OS. On the other hand, acquired mutations in *ERBB4* and *FAT4* genes were found to be significantly more hazardous than wild-type genotypes (Fig. 4B). Of the 19 patients, three had *ERBB4* mutations (P09 - p.P1283Q, P20 - p.M1? & p.I458T, P24 - p.R782G) and three had *FAT4* mutations (P15 - p.R2685Q, P19 - p.L4041I, P20 - p.P2961L). Both *ERBB4* p.P1283Q and *FAT4* p.R2685Q have been reported on the IntOGen website (www.intogen.org) as confirmed driver mutations.

Survival analysis revealed that *ERBB4* mutations (Fig. 4C) and *FAT4* mutations (Fig. 4E) were significantly associated with lower PFS in patients (*ERBB4*: *P* = 0.018, *FAT4*: *P* = 0.042). Patients without *ERBB4* mutations had a median PFS of 8.45 months while those with mutations on this gene, on average, experienced relapse after only 3.23 months (HR = 4.71, 95% CI: 1.15–19.31; *P* = 0.018) (Fig. 4B, C). A similar pattern was observed in patients with *FAT4* mutations, who, on average, saw disease progression two months earlier than those without mutations (mPFS: 8.45 vs. 6.03 months; HR = 4.01, 95% CI: 0.94–17.06; *P* = 0.042) (Fig. 4B, E). However, neither *ERBB4* mutations (mOS: 38.30 vs. 5.80 months; *P* = 0.360) (Fig. 4D) nor *FAT4* mutations (mOS: 38.30 vs. 8.27 months; *P* = 0.058) (Fig. 4F) showed significant correlation with the OS of patients. Due to the observational nature of this portion of the analysis, validation through gene knockout may be necessary to further affirm mechanistic properties of these genes in acquired trastuzumab resistance.

### Extensive branching pattern correlates with poor progression-free survival

**Fig. 5:**
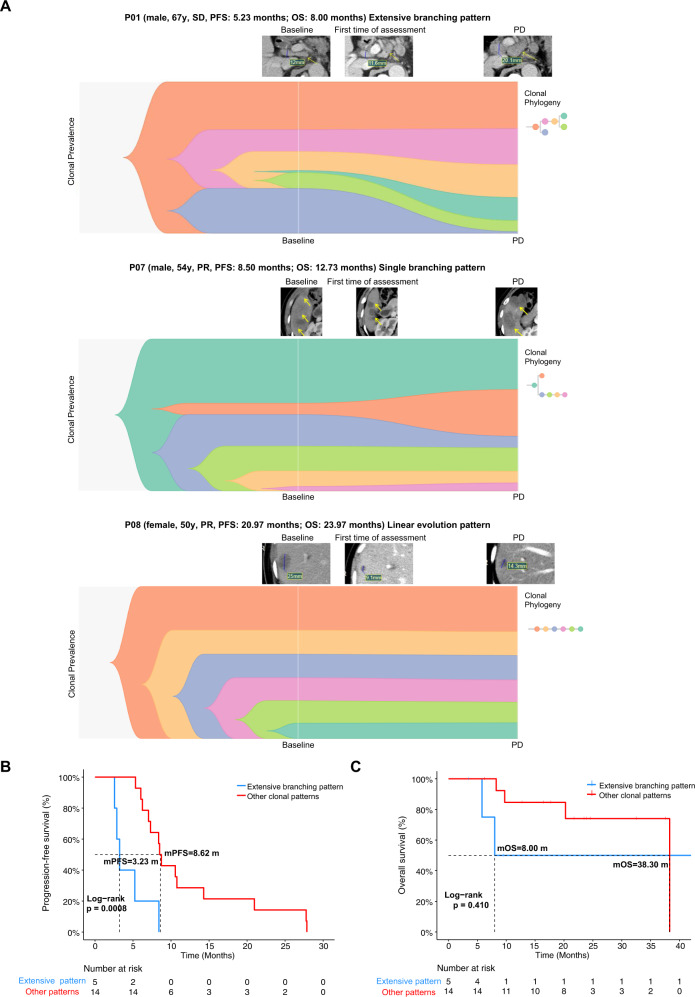
Clonal evolution pattern predicts response to progression-free survival in patients. **A** Example fishplot for patients with extensive branching, single branching, and linear evolution patterns. Radiology imaging of the tumor site in each corresponding patient was displayed in relation to the time point when the image was captured. Sex, age, best response to treatment, PFS, and OS for each patient was shown above the corresponding fishplot. **B** Hazard of extensive branching pattern over other evolutionary patterns with PFS or **C** OS as the endpoint. SD stable disease, PR partial response, PD progressive disease.

Clonal evolution patterns were shown in Fig. 5A and Supplemental Fig. S5. Six patients were identified as having a linear evolution pattern, 11 with only a single branching point, and five with an extensive branching pattern (i.e., Multiple branching points). Figure 5B shows that extensive branching has a significantly lower PFS than other branching patterns (mPFS: 3.23 vs. 8.62 months; HR = 0.16, 95% CI: 0.05–0.53; *P* = 0.0008). There was no significant difference between the groups for OS (mOS; HR = 0.47, 95% CI: 0.08–2.90; *P* = 0.410; Fig. 5C).

Max change in clonal cell fraction (CCF) was defined as the change in clonal fraction that was largest, either positive or negative, among all sub-clones in a patient. Clonal fraction was identified by Pyclone. Positive results indicate an increase in clonal fraction, while a negative change in CCF indicate a decrease in clonal fraction. We grouped patients based on whether the maximum change in CCF was positive or negative. No significant results were observed in OS or PFS of the two groups (Supplemental Fig. S6A, B).

With these results, we found that patients who exhibit extensive branching pattern have a lower PFS. Patients who seemingly acquired resistance more rapidly, tended to have a higher diversity of sub-clonal populations than patients who did not experience rapid disease progression. This may indicate that the mechanism behind acquired resistance to trastuzumab is correlated with the emergence of sub-clonal populations or may favor an environment which promotes the development of sub-clonal populations.

## Discussion

In this study, we identified potential mechanisms of acquired trastuzumab resistance as well as clinical and molecular factors for predicting primary trastuzumab resistance using paired tumor tissue samples at baseline and after PD. The acquisition of tissue biopsy samples over the more commonly used liquid biopsy samples was a crucial design choice during the conception of this study, which ensured that collected genomic sequences all belonged to the tumor site of interest. The choice to collect sample from the same tumor location over two timepoints minimized the genetic variation between samples, which allowed for us to focus on more relevant acquired mutations and CNVs.

A preliminary comparison between clinicopathological characteristics revealed that PFS was correlated with Lauren classification of tumor. Patients with intestinal subtype experiences significantly higher sensitivity to trastuzumab treatment and, thus, longer PFS than the diffuse type. A similar result was reported in a paper by Stiekema et al. published in 2013, where they observed a significant difference between overall survival of intestinal and diffuse subtype GC patients [17]. Multiple previous studies [18–20] have also suggested that patients with the intestinal Lauren classification showed significantly higher HER2 expression than the those with the diffuse subtype, meaning that treatment which target HER2, such as trastuzumab, become more effective in the intestinal subtype.

Next, we explored how CIN could be used to predict response to trastuzumab in advanced GC patients. Unexpectedly, the results of this analysis showed that patients with better response to treatment tended to have more unstable chromosomes. One study published in 2020 stated that intermediate CIN (≥50%) in breast cancer patients could indicate sensitivity to trastuzumab treatment [21]. They proposed that this correlation stems from the existence of sub-clonal populations within the tumor that originally had high CIN. The drugs may have acted on those clones specifically while low chromosomal instability clones escaped notice, thereby giving the impression of good response to treatment.

We also endeavored to identify potential genetic biomarkers that could convey primary trastuzumab resistance in patients. We discovered that *NOTCH1* deletion was present in 75% of primary resistance patients and was significantly correlated with low PFS and OS. Depending on the cancer type, *NOTCH1* can act as either a tumor suppressor gene or an oncogene [22–25], but no evidence has been shown regarding the role of *NOTCH1* deletion events in GC. Here, we would like to propose a hypothesis, where when *NOTCH1* undergoes copy number deletion, the number of *NOTCH1* proteins decreases. *NOTCH1* has been proven to act as a promoter for a *MYC* enhancer that promotes T cell development in leukemia [26]. A decrease in *NOTCH1* proteins will indirectly hinder the development of T cells, meaning that when trastuzumab treatment is administered, there are fewer T cells available to be recruited for immunogenic response.

Previous research has explored the feasibility of using NGS as a proxy for determining ERBB2 amplification status in place of IHC/FISH testing [14, 17]. In comparison to the current gold standard of IHC/FISH validation of CNVs, NGS methods have the benefit of requiring fewer samples, having a faster turnaround time, being more objective, and being able to simultaneously check multiple markers. In our analysis, we discovered that HER2 positivity was not detected in two of the 23 patients when using the NGS approach. This is in line with past papers, which have also concluded that ERBB2 amplification detection via NGS in GC is less reliable than in breast cancer [17]. This is likely due to the highly heterogeneous landscape of the former [27]. While our findings support this theory, our results show a higher sensitivity than was previously reported. This difference may be an effect of having higher coverage for the ERBB2 gene with WES instead of whole genome sequencing.

Throughout our analysis, we discovered that several gene’s copy number was altered. A review of the current literature has revealed potential mechanistic properties of MYC amplification [28, 29], STK11 deletion [30, 31], and LRP6 amplification [32] in response to treatment. In particular, a previous study examined the effect of AURKA amplification as a driver gene in GC patients [33]. The significance of this gene is that it promotes tumor cell growth and proliferation and thus, may be a mechanism of resistance for GC patients who develop resistance to trastuzumab. In addition, AURKA has been reported to be involved in the resistance of third-generation tyrosine kinase inhibitors in lung cancer patients [34, 35]. In those cases, the proposed mechanism of action involves activation of AURKA which, in turn, suppresses downstream factors that control apoptosis. Due to the similarity in targets, we hypothesize that the mechanism of acquired resistance to trastuzumab may be analogous in nature. It will be intriguing to test the function of the AURKA gene in trastuzumab resistance by introducing small molecule inhibitors in conjunction with animal studies.

We also identified two genes that were significantly correlated with PFS in 19 patients with acquired resistance. We believe that PFS was a suitable endpoint as it represents how hazardous mutations in a gene can be, and a lower PFS would indicate that acquired mutations confer resistance more rapidly. The study by Wang et al. [13] identified high-frequency ERBB2/4 mutations as a mechanism which may induce rapid acquisition of resistance to trastuzumab in advanced HER2-positive GC patients. In addition, ERBB4 has previously been reported to act as a mediator for acquired resistance against a different HER2-targeted drug, lapatinib, in breast cancer [36]. Upon inhibition of ERBB2, they deduce that ERBB4 may take over as the dominant pathway for cancer growth. Although the previous study [36] reported low levels of HER4 protein expression, they did not rule out the possibility of regulation through other means, such as post-translational modification of HER4 protein or autocrine signaling. Considering this, the proteomic environment of samples should be investigated before conclusions can be drawn. Furthermore, acquired mutations in FAT4 were found to correlate significantly with lower PFS in patients. FAT4 was thought to inhibit YAP1-mediated cell proliferation [37]. Thus, when FAT4 is inactivated through acquired mutations, inhibition of YAP1 cell proliferation is reduced and YAP1 signaling enhances the growth and invasion of cancer cells [38]. Both potential mechanisms mentioned above bypass HER2 by introducing a new pathway for cancer to progress, which may lead to the development of a variety of trastuzumab-resistant sub-clonal populations. This would explain the extensive branching pattern observed in patients with poor response to treatment. Four of the seven mutations were found in patients with extensive branching. In fact, three out of the four mutations found in ERBB4 and one out of the three mutations found in FAT4 belonged to patients who also exhibited extensive clonal branching. This strongly suggests that sub-clonal evolutionary pressures may be a novel avenue of investigation for mechanisms of acquired trastuzumab resistance.

In conclusion, we have identified several possible genetic and clinical factors at baseline that may predict primary resistance to trastuzumab. These include Lauren classification, lower CIN, and *NOTCH1* deletion. *ERBB4* and *FAT4* mutations were identified as potential mechanisms for acquired resistance to trastuzumab. Furthermore, we saw evidence of disease progression being correlated with an extensive branching pattern during clonal evolution. In our future investigations into this topic, we will attempt to mitigate the issue of sample size by increasing the time allotted for sample collection. By researching the potential mechanisms of resistance, we hope to one day influence treatment evaluations in the clinical setting, thereby helping countless patients select the most suitable treatment.

## Methods

### Study design and participants

This study was conducted at the Cancer Hospital of the University of Chinese Academy of Sciences (Zhejiang Cancer Hospital) in accordance with a human research ethics committee-approved protocol (Approval No. IRB-2022-70). The study included 24 patients with histologically confirmed GC or gastroesophageal junction cancer (GEJC) who received trastuzumab in combination with chemotherapy as the first-line treatment between April 2018 and March 2021. Informed consent was obtained from all participants. The study design is shown in Supplemental Fig. S1 and Supplemental Methods.

### Sequence data processing and mutation calling

Methods for sequence data processing and mutation calling can be found in the Supplemental Methods.

### Copy number variation analysis

Copy number variations (CNVs) were detected using FACETS [39] with default parameters. Somatic CNVs were identified using paired normal/tumor samples for each gene and copy number level was determined using past literature [40]. The resulting copy number variation list was further filtered through an internally collected list (249 genes; 113 amplifications, 136 deletions) of hot genes. Similar to the previous section, CNVs were also filtered for three or more supporting samples. Acquired CNVs were defined as variants that are present in post-disease progression samples but absent in baseline samples.

### Clonal evolution

Clonal fraction for each of the 46 samples was calculated using the Pyclone [41] tool. SCHISM [42] was used to predict sub-clonal hierarchy and evolutionary relationship. Fish plots were drawn using Timescape (v3.14). Patients with no branching in the evolutionary tree were grouped as the “Linear” evolution pattern. Patients that had one branch point were classified as the “Single branching” evolution pattern. Patients with more than one branch point were classified as the “Extensive branching” evolution pattern.

### Statistical analysis

Statistical analyses were performed with R 3.5.2. Quantitative data were presented as median (range) or the number of patients (percentage) unless otherwise indicated. Between‐group differences were analyzed using the Fisher’s exact test. Two comparison categories were used for Fisher’s test. We considered comparisons between responders (complete response (CR) + partial response (PR)) versus (vs.) nonresponders (Progressive disease (PD) + Stable disease (SD)) or acquired resistance (CR + PR + SD) vs. primary resistance (PD). Survival analysis was performed using the Kaplan–Meier method, p values were determined with the log-rank test, and hazard ratios (HRs) were calculated by Cox proportional hazards. Univariate analysis was performed using the Cox proportional hazard regression model to determine the associations between different variables and PFS or OS. A significant *P* value was set at <0.05. Kaplan–Meier survival curves are generated using the “survival” package (v3.2-13) and “survminer” package (v0.4.9). Wilcoxon ranked sum test was used for determining the significance between boxplots. Jonckheere’s trend test was used to determine significant trends in data.

In this study, tumor mutation burden (TMB) was defined as the total number of non-synonymous mutations divided by the total length of the sequences (in Mb). Samples were sorted from low to high TMB and patients in the lower third were classified as “low TMB”, on the other hand, the remaining patients in the higher two-thirds were classified as “high TMB”. Chromosomal Instability (CIN) was defined as the proportion of the genome that have aberrant copy numbers (i.e., segment-level copy number ≤1 or ≥3).

## Supplementary information

Supplemental table S1

Supplemental table S2

Supplemental table S3

SUPPLEMENTAL MATERIAL

## Data Availability

Due to local restrictions on the sharing of genomic information, the data that support the findings of this study are available on request from the corresponding author.
