# Supplementary material for: Exploring potential molecular resistance and clonal evolution in advanced HER2-positive gastric cancer under trastuzumab therapy
Source: Oncogenesis. 2023 Apr 18;12(1):21. doi: 10.1038/s41389-023-00466-2 (PMC10113330; doi:10.1038/s41389-023-00466-2)
Supplement: Supplementary file 4 — SUPPLEMENTAL MATERIAL [file 41389_2023_466_MOESM4_ESM.docx]

**Supplemental Information for:**

**“Exploring potential molecular resistance and clonal evolution in advanced HER2-positive gastric cancer under trastuzumab therapy”**

**Submitted to *Oncogenesis***

Co-corresponding author:

* Dr. Jieer Ying

Department of Hepato-Pancreato-Biliary & Gastric Medical Oncology, Cancer Hospital of the University of Chinese Academy of Sciences (Zhejiang Cancer Hospital), Hangzhou, 310022, China

Phone Number: +86 13858195803

Email: jieerying@aliyun.com

* Dr. Jianying Jin

Department of Medical Oncology, Taizhou Hospital of Zhejiang Province, 317000, China

Phone Number: +86 13867656027

Email: 302861459@qq.com

**Table of contents:**

Supplemental Methods

Supplemental Figure S1

Supplemental Figure S2

Supplemental Figure S3

Supplemental Figure S4

Supplemental Figure S5

Supplemental Figure S6

Supplemental table legends

References

**Supplemental Methods**

**Study design and participants**

Patients were enrolled into this study if a patient had (1) clear tissue histology of unresectable metastatic, or late-stage GC, (2) received trastuzumab combined chemotherapy as first line therapy, (3) an immunohistochemistry (IHC) status of IHC 3+ or IHC 2+ with fluorescence in-situ hybridization (FISH) positive (FISH+), and (4) have tumor tissue sample obtained at PD after treatment. Patients were rejected from this study if (1) tissue samples were unable to be obtained at PD after trastuzumab treatment, (2) patients received multi-line therapy between the two biopsies, or (3) if trastuzumab treatment was combined with other anti HER2 treatment therapies. All patients provided written consent for sample collection and research intent before enrollment. Clinicopathological data, including age, sex, Eastern Cooperative Oncology Group performance status (ECOG PS), Lauren classification, differentiation, number of metastasis sites, organ(s) of metastasis, and initial HER2 status verification were retrospectively reviewed. Primary resistance patients were identified as having PD as best overall response (BOR) to treatment. Response to treatment was determined according to RECIST v1.1.

**Sample collection and preprocessing**

Paired tumor tissue samples were collected from 24 stage IV GC patients both at baseline and at the time of PD. The tumor locations were obtained from the gastroesophageal junction (GEJ) in 4 patients (16.6%), and gastric body in 20 patients (83.4%). HER2 status was identified using IHC and validated using FISH(1). Normal blood samples were also collected and used as the normal control to remove germline variations. Tumor tissue samples were processed and sequenced in similar fashion as described in the paper by Wang et al(2). The samples underwent whole exome sequencing (WES) on Illumina Hiseq 4000 platform in a centralized clinical testing center (Nanjing Geneseeq Technology Inc.).

**Sequence data processing and mutation calling**

Leading/trailing low quality (quality reading below 30) or N bases were removed with fastp (v0.20.0). Reads from each sample were mapped to the reference sequence hg19 (Human Genome version 19) using Sentieon (v0.7.12). Local realignment around indels and base quality score recalibration was applied with the Genome Analysis Toolkit (GATK 3.6).

Mutect2 (v4.1.2.0) was employed for detection of candidate somatic mutations in tissue samples. We filtered for mutations that had a minimum variant allele frequency of 2% with at least 5 variant supporting reads. Mutations were also removed if they were present in >1% population frequency in the 1000 Genomes Project or 65000 exomes project (ExAC). Only mutations that appear in three or more samples were retained for survival analysis. Mutations that appeared in the PD samples but was absent in baseline samples were selected as acquired mutations.

**Supplemental Figures**


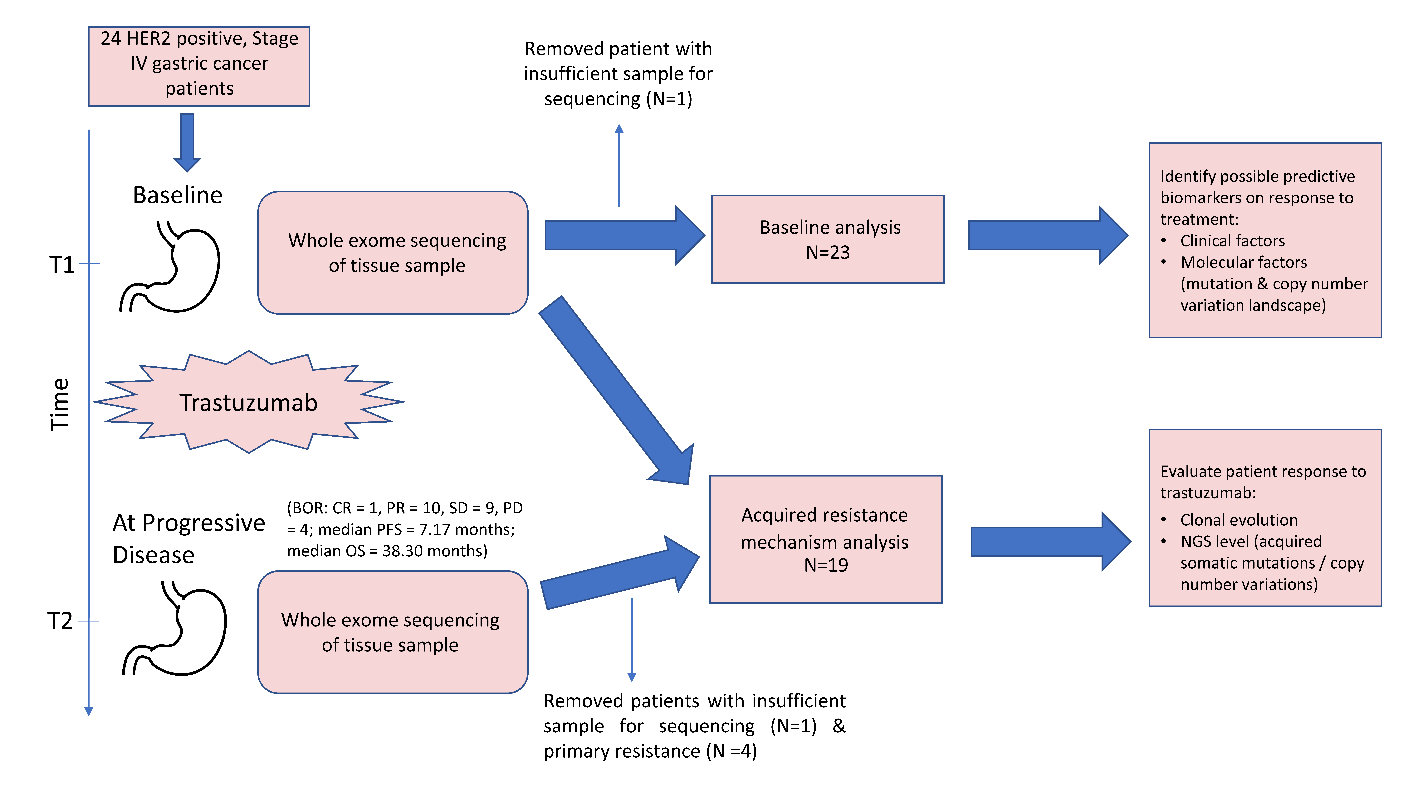


**Supplementary figure S1. Study design for molecular analysis.** Consent was received from 24 patients with stage IV HER2-positive GC. All patients underwent treatment with trastuzumab. Samples were collected both before treatment and at PD. WES was performed on samples from 23 patients. Mutation and copy number variation data was used for further analysis. BOR: Best overall response, CR: Complete response, PR: Partial response, SD: Stable disease, PD: Progressive disease, PFS: Progression free survival, OS: Overall survival.


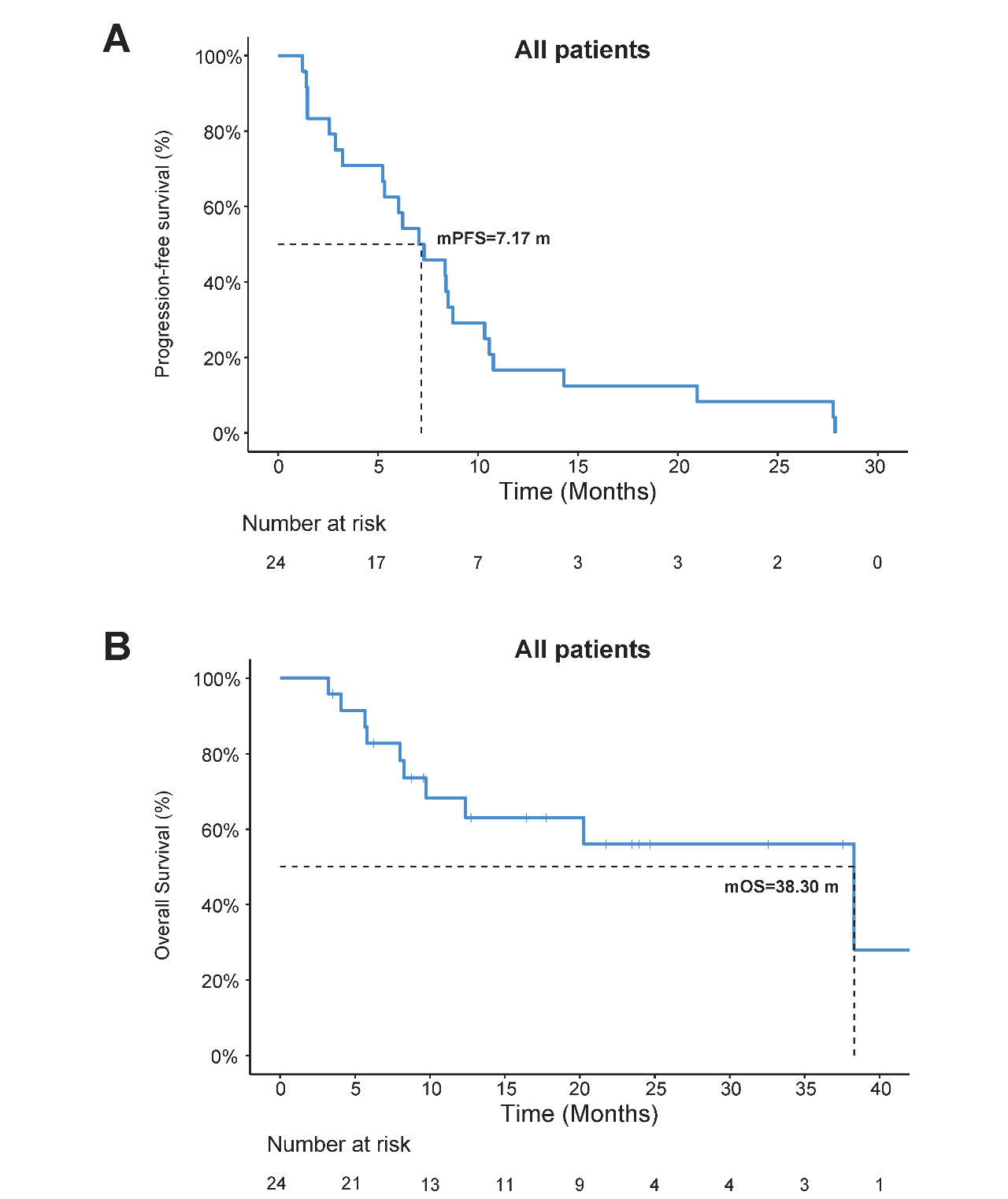


**Supplementary Figure 2.** **Kaplan-Meier survival curve for all patients based on PFS or OS.** **A)** PFS and **B)** OS for all 24 patients.


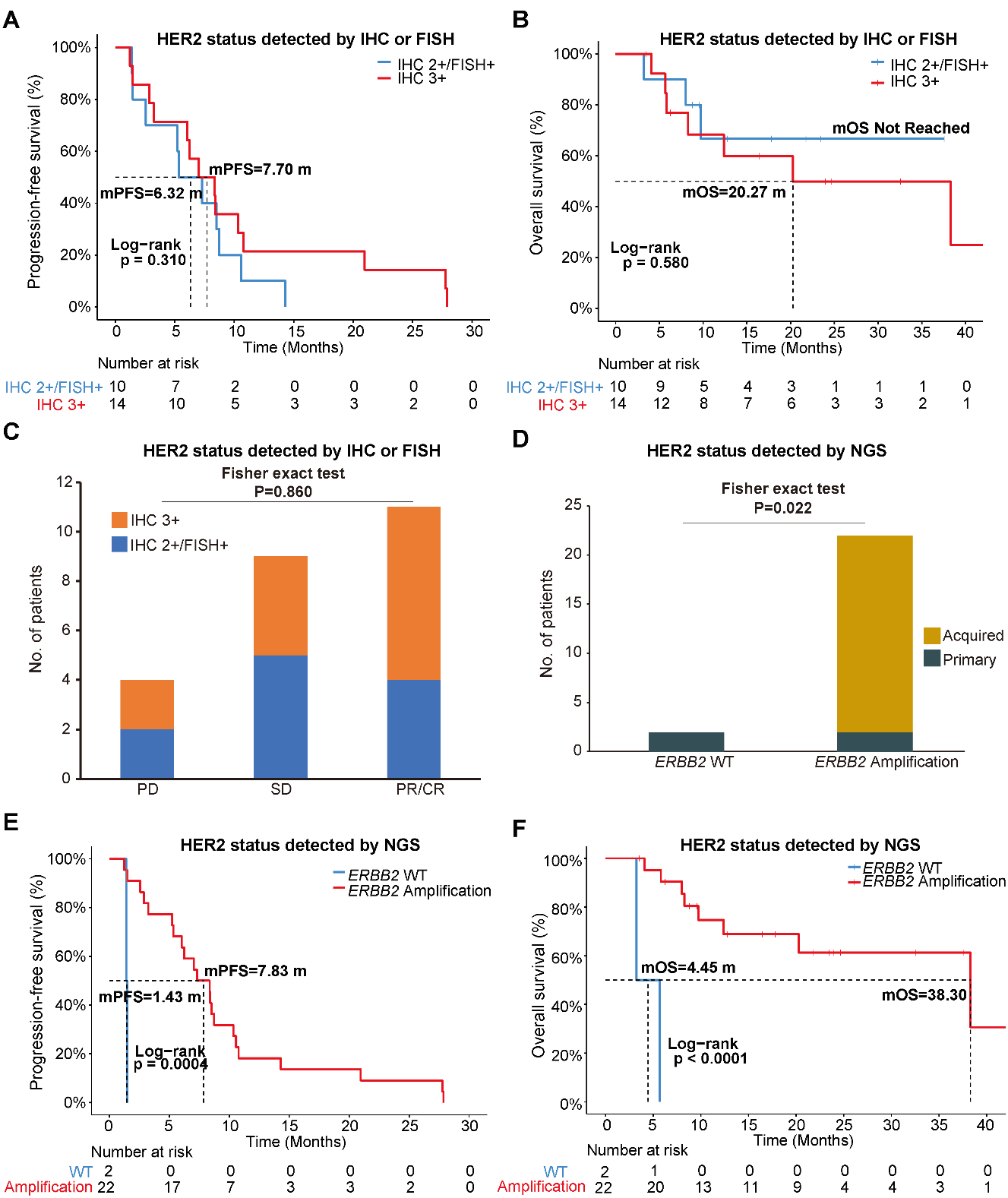


**Supplementary Figure 3. Comparison of HER2 status as determined by IHC/FISH and NGS at baseline. A)** Survival curve of IHC 3+ vs. IHC 2+/FISH+ groups with PFS or **B)** OS as endpoint. **C)** Distribution of IHC 3+ and IHC 2+/FISH+ patients in each response category. Fisher’s exact test comparison with Freeman-Halton extension was used to determine the significance of all three categories simultaneously. **D)** Number of DCR and PD patients classified as *ERBB2* amplification or WT determined by NGS methods. Fisher’s exact test was used to determine significance. **E)** Survival curve of *ERBB2* amplification vs. WT groups with PFS or **F)** OS as the endpoint. IHC: Immunohistochemistry, FISH: Fluorescence in-situ hybridization.


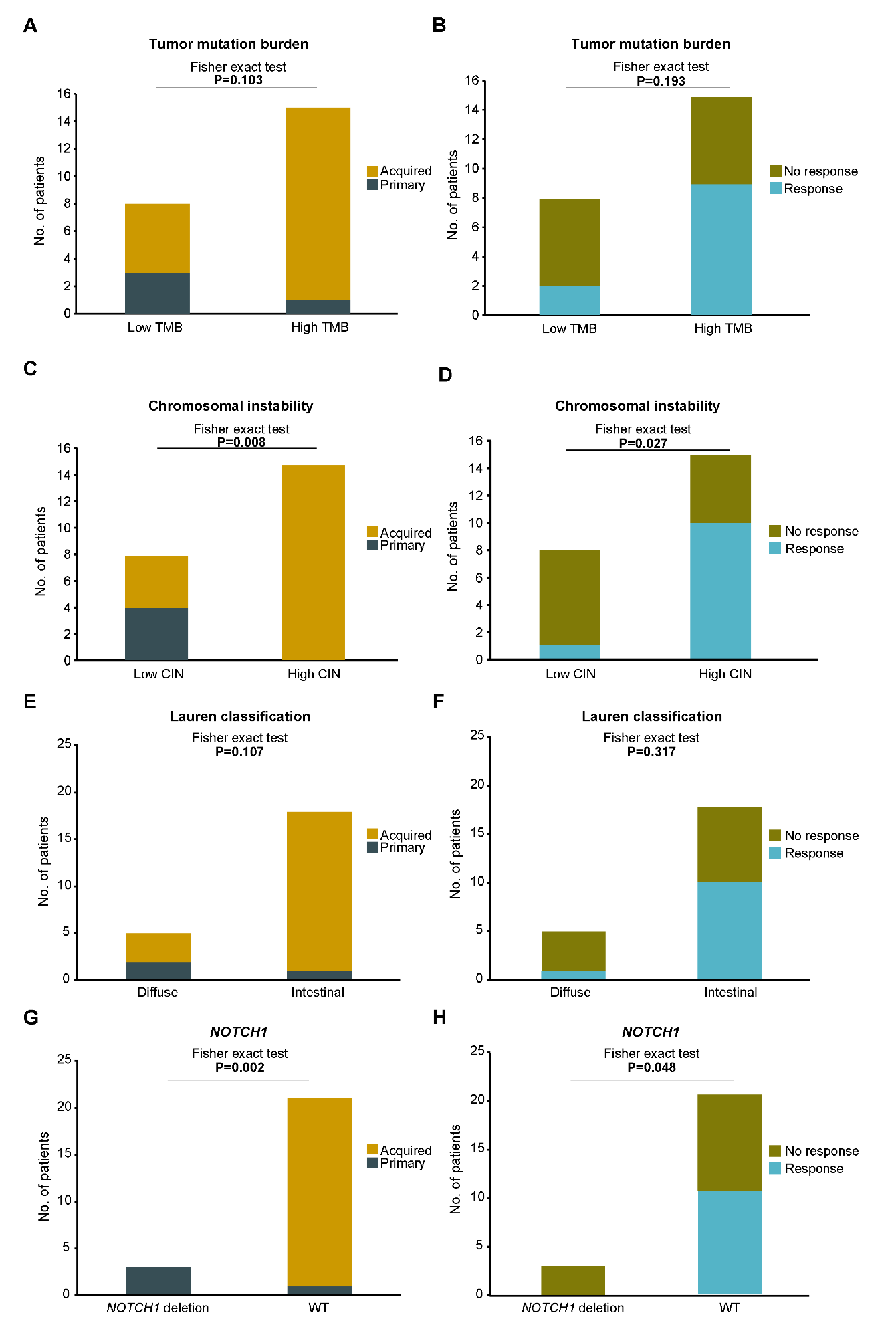


**Supplementary Figure 4.** **Correlation of high/low TMB, high/low CIN, and *NOTCH1* deletion/wild-type patients with different response categories**. **A)** Distribution of high TMB and low TMB patients as classified by primary resistance or acquired resistance. **B)** Distribution of high TMB and low TMB patients as classified by response (CR+PR) or no response (SD+PD) to treatment. **C)** Distribution of high CIN patients (≥50%) and low CIN (<50%) patients as classified by primary resistance or acquired resistance. **D)** Distribution of high CIN patients and low CIN patients as classified by response or no response. **E)** Distribution of diffuse Lauren classification patients and intestinal classification patients as classified by primary resistance or acquired resistance. **F)** Distribution of diffuse Lauren classification patients and intestinal classification patients as classified by response or no response. Mixed Lauren classifications were not included in this portion of analysis. **G)** Distribution of patients with *NOTCH1* deletion and WT as classified by primary resistance or acquired resistance. **H)** Distribution of patients with *NOTCH1* deletion and WT as classified by response or no response. Fisher’s exact test was used to determine significance of proportions. CR: Complete response, PR: Partial response, SD: Stable disease, WT: Wild-type, TMB: tumor mutation burden, CIN: Chromosomal instability.


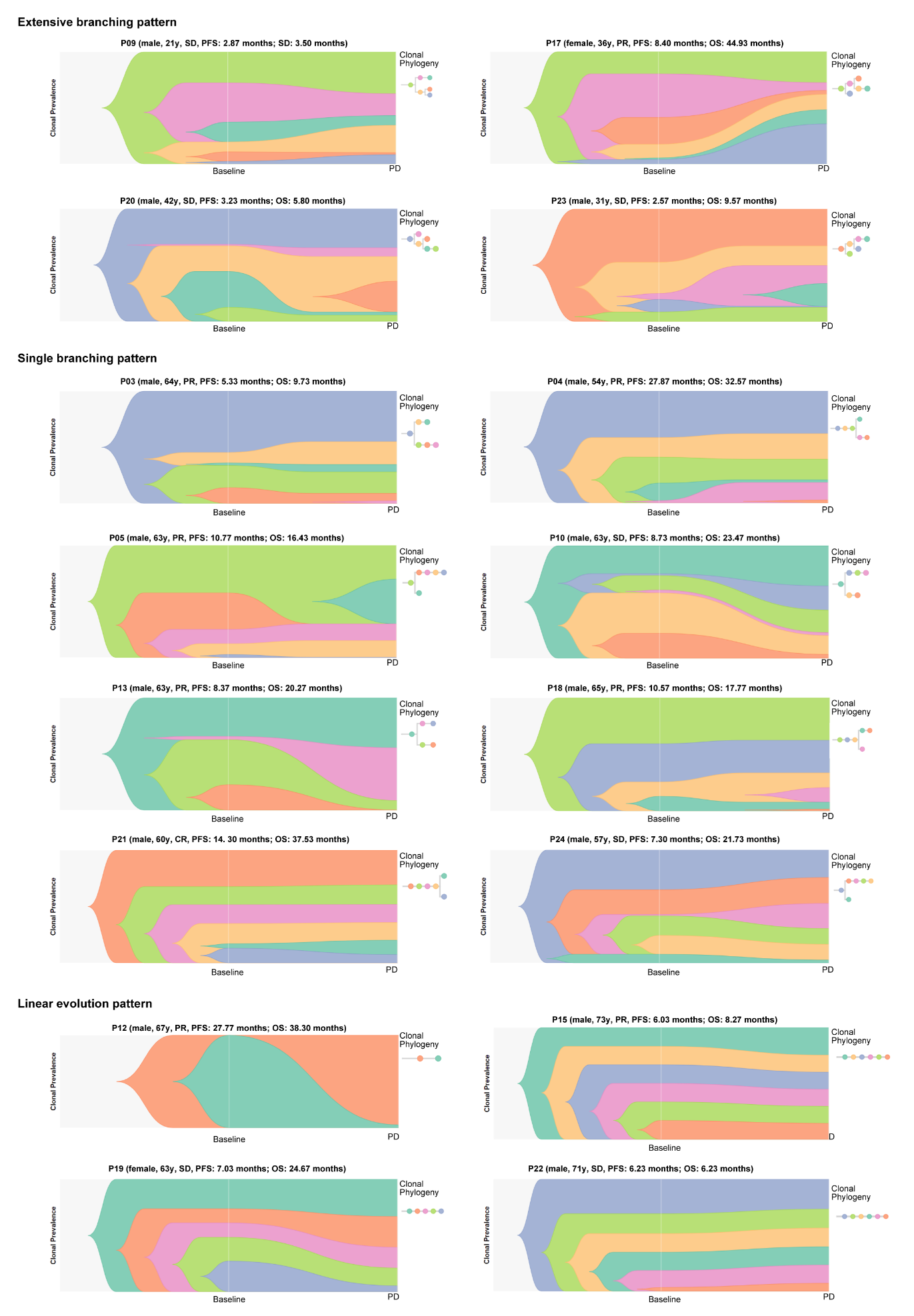


**Supplementary Figure 5. Evolutionary patterns of the other 16 patients.** Fishplots were grouped according to whether they exhibit extensive branching, single branching, or linear evolutionary patterns. Sex, age, best response to treatment, PFS, and OS for each patient was shown above the corresponding fishplot. CR: Complete response, PR: Partial response, SD: Stable disease, PD: Progressive disease, PFS: Progression free survival, OS: Overall survival.


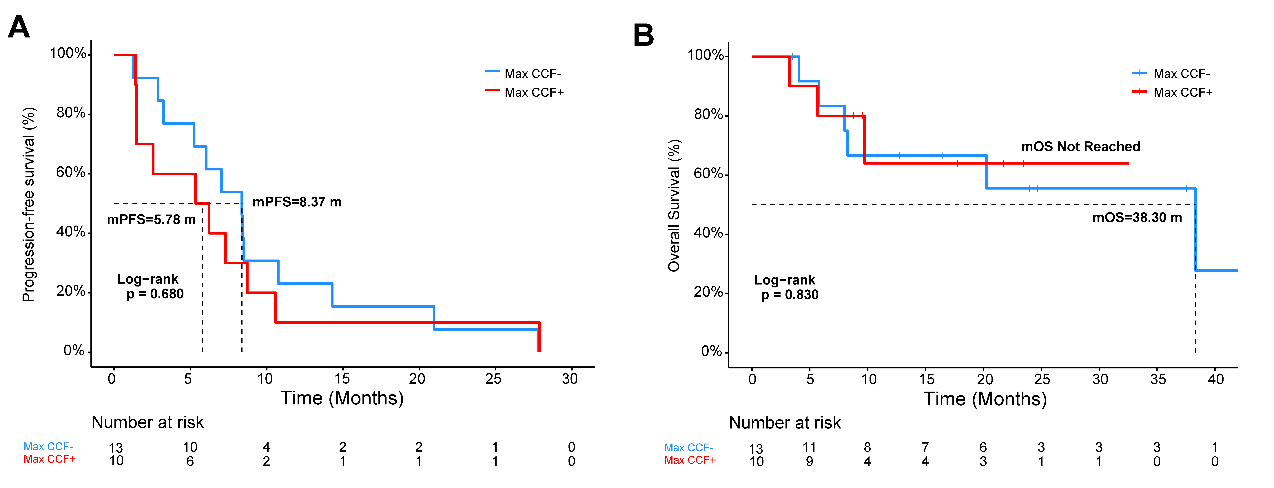


**Supplementary Figure 6. Correlation of positive or negative max change in CCF with survival.** **A)** PFS or **B)** OS for both categories of CCF change. Patients were classified according to whether max change in CCF for that patient was negative (“Max CCF-”) or positive (“Max CCF+”). CCF, Clonal cell fraction.

**Supplemental table legends**

**Supplementary table S1.** Association analysis between clinical factors or molecular factors and response, PFS or OS.

**Supplementary table S2.** Univariate Cox regression analysis of PFS or OS with individual gene mutation at baseline.

**Supplementary table S3.** Univariate Cox regression analysis of PFS or OS with individual gene copy number variations at baseline.

**References**

1. Wolff AC, Hammond MEH, Allison KH, Harvey BE, Mangu PB, Bartlett JMS, et al. Human Epidermal Growth Factor Receptor 2 Testing in Breast Cancer: American Society of Clinical Oncology/College of American Pathologists Clinical Practice Guideline Focused Update. J Clin Oncol. 2018;36(20):2105-22. Epub 20180530. doi: 10.1200/JCO.2018.77.8738. PubMed PMID: 29846122.

2. Wang DS, Liu ZX, Lu YX, Bao H, Wu X, Zeng ZL, et al. Liquid biopsies to track trastuzumab resistance in metastatic HER2-positive gastric cancer. Gut. 2019;68(7):1152-61. Epub 20180929. doi: 10.1136/gutjnl-2018-316522. PubMed PMID: 30269082.
